# Supplementary material for: Respiratory Syncytial Virus Matrix Protein-Chromatin Association Is Key to Transcriptional Inhibition in Infected Cells
Source: Cells. 2021 Oct 18;10(10):2786. doi: 10.3390/cells10102786 (PMC8534903; doi:10.3390/cells10102786)

Supplementary Materials

Supplementary Figure S1. RSV M's ability to inhibit transcription *in vitro* is dependent on R170/K172. (**A**) Increasing amounts of purified His-Mwt or His-M-R170T/K172T protein were added to 3.5 ng DNA template (pGEM Express Positive Control Template, 96 pM) for 15 min at room temperature and then added to a cell-free T7 transcription assay (Riboprobe System T7 kit). Samples were incubated for 1 h at 37C, run on a 1% agarose gel with ethidium bromide and imaged under UV illumination. (**B**) Levels of transcribed RNA from (**A**) were quantified using the NIH ImageJ software, and expressed as a percentage of the levels of transcript in the absence of M. The concentrations of the respective proteins inhibiting transcription by 50% (K_i) estimated from the curves are indicated.

Supplementary Figure S2. R170/K172 residues are not critical for M nuclear export. Overnight cultures of Vero cells grown on coverslips were transfected to express GFP-Mwt or GFP-M-R170T/K170T using Lipofectamine 2000 (1:1 ratio of DNA to reagent). 18 h after transfection, cultures were treated either with or without leptomycin B (LMB). 20-24 h after transfection the localisation of GFP fusion proteins was assessed by quantitative live cell CLSM, with cells transfected to express GFP alone or GFP-Rev (positive control for LMB action) as controls (typical cell images shown above). Analysis for the extent of nuclear accumulation (Fn/c) was performed as previously [14, 45, 46]. Data represent the mean $\pm$ SEM ($n \geq 30$) and are representative of a series of 3 similar experiments. * $p < 0.05$, *** $p < 0.001$.

Supplementary Figure S3. The R170/K172 residues are not critical for M RNA binding *in vitro*. Increasing concentrations of purified His-Mwt or His-M-R170T/K172T protein were

incubated with 200 ng RNA for 15 min at room temperature, and the protein-RNA complexes electrophoresed on an 0.8% agarose gel for 8 h at 4C followed by staining with ethidium bromide. Levels of unshifted (unbound) RNA were quantified using the NIH ImageJ software, with results expressed as a percentage of the value in the absence of M protein. Association constants (Kas) estimated from the curves are indicated.

Supplementary Figure S4. Recombinant RSV carrying arginine 170/lysine 172 mutations shows impaired virus replication. Vero cells were infected with wild type rA2 (white columns) or mutant (rA2-M:R170T/K172T; black columns) at an MOI of 1. Cells were harvested at the times indicated p.i. and total RNA isolated. RNA was used to determine virus RNA genome equivalents by RT-qPCR by extrapolation from a standard curve produced from results using a plasmid carrying the RSVA N gene cDNA. Results represent the mean + SEM (n = 3), from a series of 2 similar experiments. * $p < 0.05$.

Supplementary Figure S1

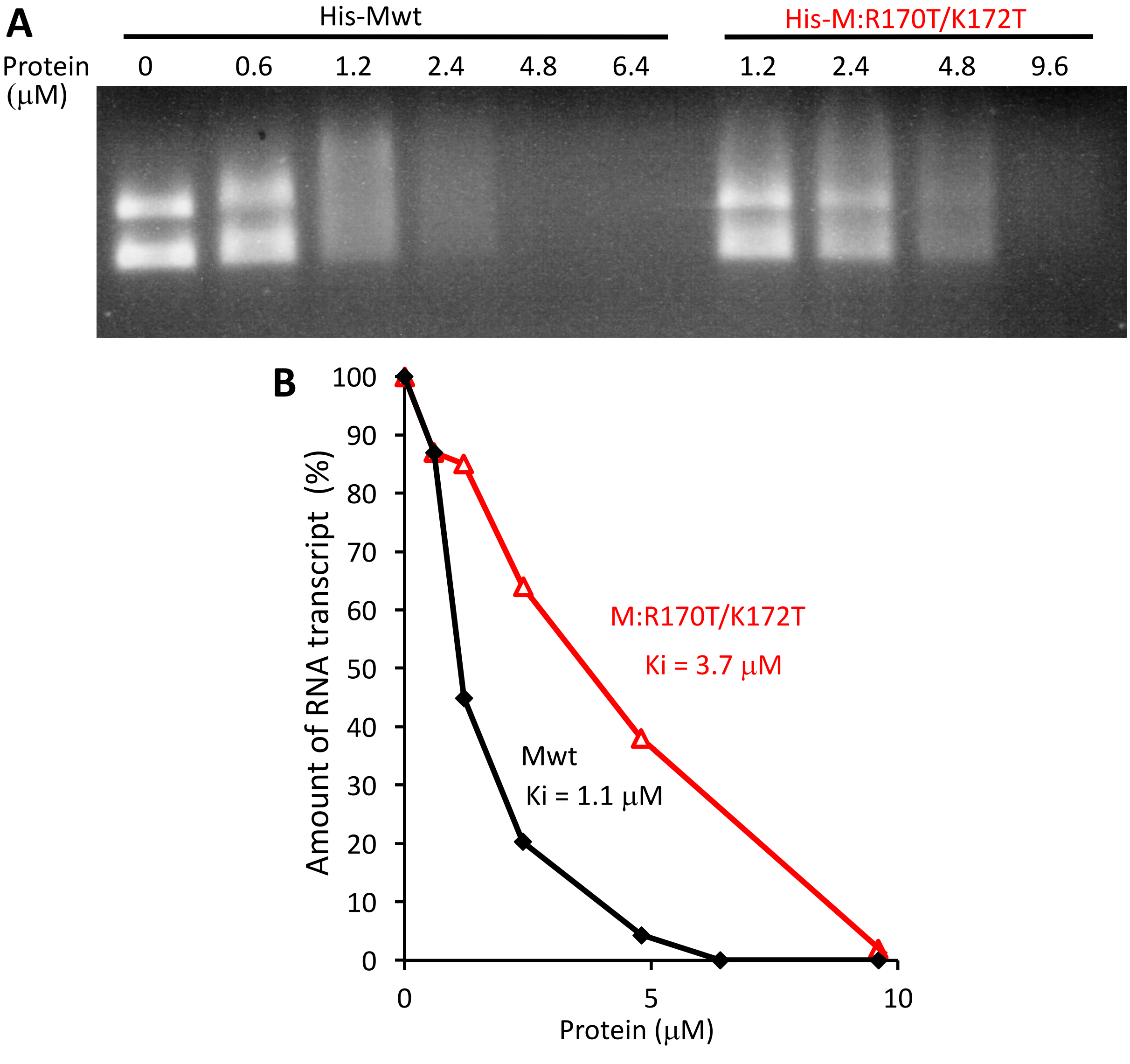

Supplementary Figure S2

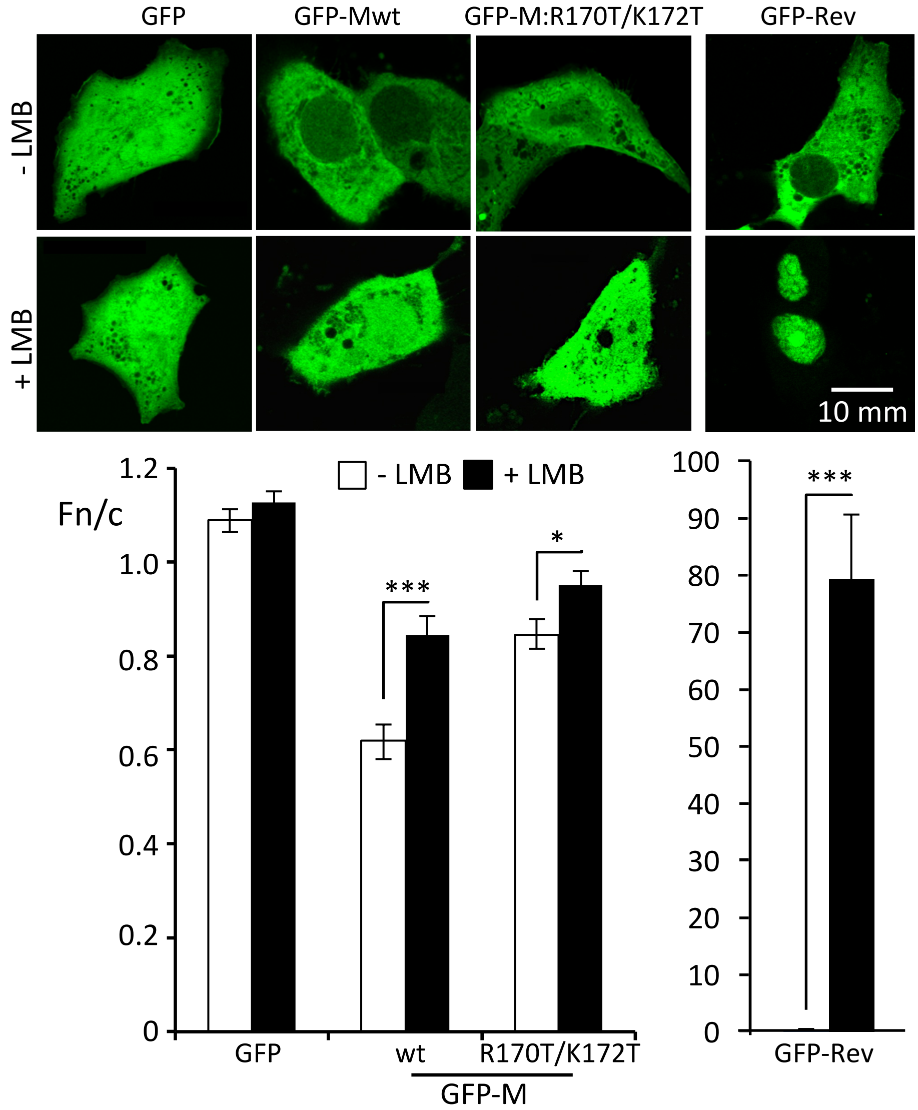

Supplementary Figure S3

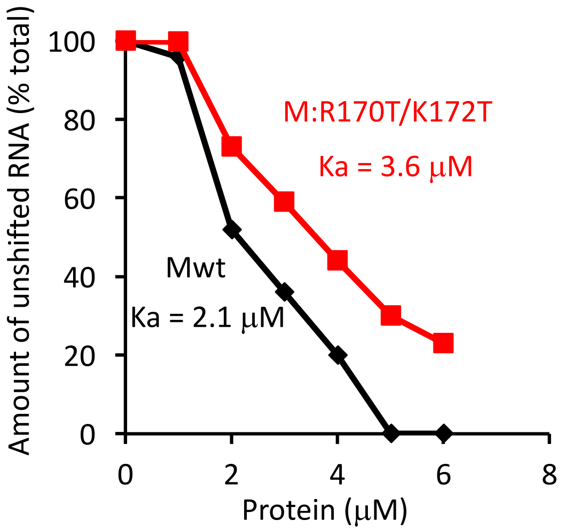

Supplementary Figure S4

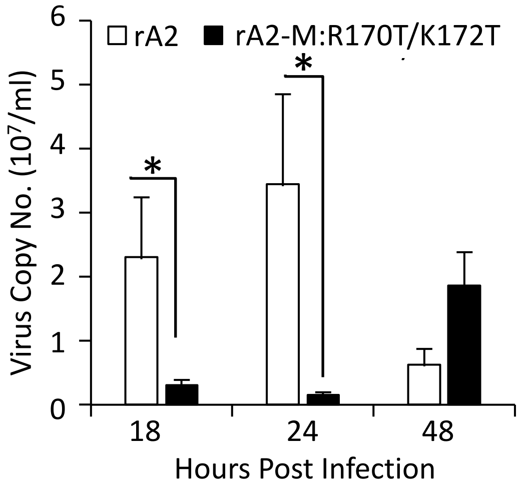

Supplement: Supplementary file 1 [file cells-10-02786-s001.zip › cells-1335954-supplementary.pdf]
